# Positive and negative contexts predict duration of pig vocalisations

**DOI:** 10.1038/s41598-019-38514-w

**Published:** 2019-02-14

**Authors:** Mary Friel, Hansjoerg P. Kunc, Kym Griffin, Lucy Asher, Lisa M. Collins

**Affiliations:** 1 School of Biological Sciences, Queen’s University Belfast, Medical Biology Centre, Belfast, UK; 2 School of Animal Rural & Environmental Sciences, Nottingham Trent University, Nottingham, UK; 3 Centre for Behaviour and Evolution, Institute of Neuroscience, Newcastle University, Newcastle upon Tyne, UK; 4 Faculty of Biological Sciences, University of Leeds, Leeds, UK; 5 School of Life Sciences, University of Lincoln, Lincoln, UK

## Abstract

Emotions are mental states occurring in response to external and internal stimuli and thus form an integral part of an animal’s behaviour. Emotions can be mapped in two dimensions based on their arousal and valence. Whilst good indicators of arousal exist, clear indicators of emotional valence, particularly positive valence, are still rare. However, positively valenced emotions may play a crucial role in social interactions in many species and thus, an understanding of how emotional valence is expressed is needed. Vocalisations are a potential indicator of emotional valence as they can reflect the internal state of the caller. We experimentally manipulated valence, using positive and negative cognitive bias trials, to quantify changes in pig vocalisations. We found that grunts were shorter in positive trials than in negative trials. Interestingly, we did not find differences in the other measured acoustic parameters between the positive and negative contexts as reported in previous studies. These differences in results suggest that acoustic parameters may differ in their sensitivity as indicators of emotial valence. However, it is important to understand how similar contexts are, in terms of their valence, to be able to fully understand how and when acoustic parameters reflect emotional states.

## Introduction

Emotions are mental states occurring in response to external or internal stimuli which influence an individual’s fitness^1^. In the dimensional model of emotions, emotional states can be mapped in two-dimensional space, where valence (positive versus negative) and arousal (intensity) characterise each dimension^1,2^. Emotional states in non-human animals have been demonstrated in a broad range of species across the taxonomic scale (e.g. insects:^3,4^; birds:^5,6^; mammals:^7,8^). However, clear indicators of positively valenced emotion are rare, because identifying consistent correlates on the valence dimension is difficult^9,10^. Emotions are multi-componential and each component can vary across the two dimensions of arousal and valence^1^. The four pillars of emotion assessment in humans are physiological, behavioural, cognitive and subjective verbal report^11,12^. Quantifying emotions in non-human animals relies on indirect assessment of three of these pillars: physiology, behaviour and cognition; the fourth pillar is solely accessible in humans. Cognitive bias testing assesses the effect of emotion on cognition in animals^13–15^. However, this test requires lengthy specialised training of animals to implement, which itself could potentially alter the emotional state of the indivudals being assessed^16,17^. Vocalisations, however, can be reliably measured and may allow the quantification of emotions in non-human animals as they reflect the inner state of the caller, providing information about an individuals’ affective state^10,18,19^. Thus, emotions expressed in vocalisations may play an important role in shapping social interactions among indiviuduals.

Morton’s motivational-structural rules state that acoustic characteristics of vocalisations are predictable from the context in which they are produced^20^. Vocalisations produced in more ‘hostile’ contexts are expected to have lower frequencies and longer durations than vocalisations produced in ‘friendly’ or ‘fearful’ contexts^20^. However, in addition to between-context variation in acoustic characteristics, variation within vocalisation types may also be context-dependent, and may reflect the internal state of the caller^21–23^. In the domestic pig, the most common vocalisation is the grunt, which functions predominantly as a contact call^24,25^. Information encoded in grunts includes body size^25^, individual identity^26^, personality type^27^ and emotional state^28^. Pigs’ vocal responses to mental and physical stressors differ depending on the nature of the stressor^29^. The cognitive bias paradigm has been applied to assess of the effects of various factors on emotional valence in pigs^7,30,31^.

The aim of this study was to test whether vocalisations produced in oppositely valenced contexts differ in their acoustic characteristics. We based the choice of acoustic parameters to measure on previous studies (see Table 1) and measured these in grunts produced in two different situations. In the first situation, the valence of the context was experimentally manipulated using cognitive bias positive and negative training trials. In the second situation, response to the probe in ambiguous test trials provided the measure of the valence of the context for the individual. The valence of each context was defined as approaching a stimulus in positive contexts and avoiding a stimulus in negative contexts^32^. We then analysed the effect of optimistic versus pessimistic responding on the acoustic characteristics of grunts, within each situation, in two separate analyses.

**Table 1:** Overview of the acoustic parameters measured and the underlying rationale for each parameter included in the analysis.

Variable	Rationale	Selected references
Duration	Expected to be longer in negative compared to positive contexts, longer duration may facilitate communication of more urgent negative emotions	^36,50^, *for review see*^10^
Mean F0	Increased tension of vocal muscles during negative emotion may cause higher F0 in negative contexts than positive contexts	^58^
AMextent	Less variability due to increased amplitude modulations in negative contexts compared to positive contexts	^37^
AM rate	Found to be higher in calls from negative contexts compared to calls from positive contexts	^22^
% Time Max Intensity	Rarely investigated parameter, increased subglottal pressure during negative emotion may increase percent time intensity is maximum	
Q25	Found to be lower in grunts produced in negative context than in a positive context	^28^
Q50	Found to be lower in grunts produced in negative context than in a positive context	^28^
Q75	Increased tension of vocal muscles during negative emotion may cause higher Q75	^37^
HNR	Can convey information about emotional arousal, decreased in grunts during higher arousal	^42^
F1mean	Found to be higher in grunts produced in negative contexts compared to positive contexts	^22^
F1 range	Found to be higher in calls produced in negative contexts compared to positive contexts	^22^

Parameter descriptions from Briefer (2012): Duration = duration of the call; F0 mean = mean fundamental frequency across the call; AM extent = mean peak-to-peak variation of each amplitude modulation; AM rate = Number of complete cycles of amplitude modulation per second; % Time Max Intensity = Percentage of total call duration when intensity is maximum; Q25 = frequency value at the upper limit of the first quartile of energy; Q50 = frequency value at the upper limit of the second quartile of energy; Q75 = frequency at the upper limit of the third quartile of energy; Harmonic-to-noise ratio (HNR) = ratio of amplitude peaks of detectable harmonics to noise threshold; F1 mean = mean frequency value of the first formant; F1 range = difference between the minimum and maximum F1 frequencies.

## Methods

### Animals and housing

We studied crossbred PIC 337, Large White x Landrace pigs, allocated into groups of 18 per pen, balanced for weight and sex, where they remained from 4–10 weeks of age at a research farm (Agri-Food and Biosciences Institute, Hillsborough, Northern Ireland). All test subjects were 10 weeks old at the time of cognitive bias testing. The experiment consisted of three replicates. Within each replicate there were two types of housing environment treatment; a barren environment and an enriched environment (cf.^27,33^ and see electronic supplementary material for full details). The barren environment had a partially slatted concrete floor and the minimum legal space allowance of 0.41 m^2^ per pig, whereas the enriched environment had a solid floor with straw bedding and a greater space allowance of 0.62 m^2^ per pig.

### Ethical note

Ethical approval for this research was granted from University of Lincoln’s College of Science Ethics Committee (COSREC62, 8/9/15). All procedures conformed to the ASAB/ABS Guidelines for the Use of Animals in Research.

### Cognitive bias testing

Twenty-seven pigs were tested in a spatial cognitive bias task (see Supplementary Methods for full details of training). On the cognitive bias test day, each pig was exposed once individually to each of the ambiguous probe locations: near positive (NP), middle (M) and near negative (NN), with pseudo-randomization between two positive (P) and negative (N) training trials resulting in 9 trials per test (i.e. P, N, M, N, P, NN, N, P, NP). Ambiguous locations were unrewarded but in positive and negative training trials the locations were baited as in training. Latency to reach the bowl was recorded in all trials. Individuals were given 30 seconds to approach the bowl and if they did not approach in that time they were recorded as having a latency of 30 sec and returned to the start box for the next trial.

The raw latency to reach the probe data violated the assumptions of the linear models, therefore we classified responses to the probe within each as “optimistic”, “pessimistic” or “neutral” (cf.^31^). Pigs’ responses in each trial were classified in relation to the mean speed of approach to the trained positive and negative targets from the final training session using the following formula:

$$adjusted\,score=\frac{x}{[(y+z)/2]}\,\times 100$$

where *x* = latency to contact the bowl during the cognitive bias test, *y* = mean latency to contact the bowl during the positive training trials, *z* = mean latency to contact the bowl during the negative training trials. As per Carreras *et al*. (2016), adjusted scores were calculated as percentages; if the adjusted score was ≤75% the response was classified as “optimistic”; if the score was >75 and <125% the response was classified as “neutral”; and if the score was ≥125% the response was classified as “pessimistic”.

### Acoustic recording and analysis

Vocalisations produced during the cognitive bias test were recorded at a distance of 1–4 meters using a Sennheiser ME66/K6 (Sennheiser Electronic GmbH & Co. KG, Wedemark, Germany) directional microphone connected to a Marantz PMD660 (D&M Professional, Kanagawa, Japan) solid state audio recorder (.wav format, sampling frequency: 44.1 kHz, resolution: 16 bit). The microphone was placed on one corner of the arena at a height of 1.5 m and pointed into the test arena (see Fig. S1 for microphone location above test arena). The recorder was switched on at the start of the test session for each individual and all trials were recorded.

**Figure 1:**
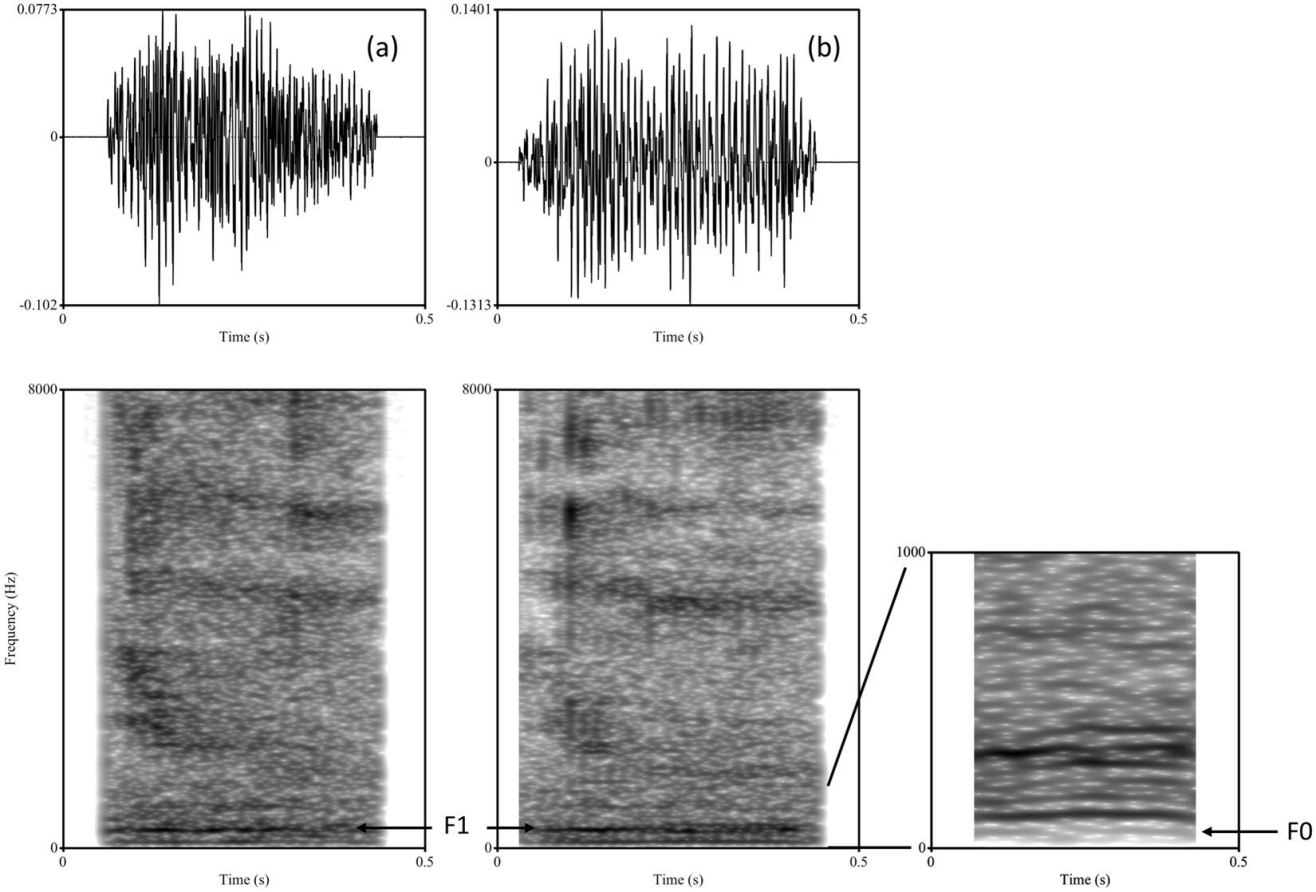
Grunt vocalisations of domestic pig. Grunt vocalisations from individual 71: (**a**) is from a positive trial with duration = 0.372 seconds and (**b**) is from a negative trial with duration = 0.411 seconds. F0 = fundamental frequency and F1 = the first formant (calls are available as audio files “Positive context grunt” and “Negative context grunt” respectively in the supplementary material). Visualisation settings: view range = 0–8000 Hz, window length = 0.03 sec, dynamic range = 65 dB, time steps = 700, frequency steps = 250, Gaussian window.

All good quality grunts with a high signal-to-noise ratio from the positive, negative and ambiguous trials were selected for analysis (see Fig. 1 for spectrograms). Grunts were chosen for analysis on the condition that they were at least three calls apart, if produced in a bout, to avoid pseudoreplication. A total of 125 calls were produced when a subject responded optimistically; 153 calls were produced when a subject responded pessimistically; 4 calls were produced when the subject’s response was neutral and these 4 calls were excluded from subsequent analyses. A further 14 calls that had been produced in positive and negative trials where the subject had responded ‘incorrectly’ (i.e. responded pessimistically in the positive trial and optimistically in the negative trial) were also excluded. This resulted in a total of 264 calls from 23 individuals for analysis (78 calls from ambiguous test trials and 186 calls from the positive and negative training trials: see Table S1 for full details of number of calls contributed per individual). Using a custom built script in PRAAT^34^, we measured 11 acoustic parameters from each call (see Table 1 for list of parameters measured and supplement for further details of acoustic analysis). Some parameters (amplitude modulations and rate) could not be measured in every call and this resulted in some missing values, hence the sample size differs between acoustic parameters (Table 2).

**Table 2:** Raw means and SDs, along with results from models for the effect of training trial type, i.e. Negative or Positive, controlled for sex, environment, and individual identity along with statistical results, sample size (*N*) and *P* values.

Row No.	Parameter	1. Trained positive/negative cue trials				${{\rm{\chi }}}_{1}^{2}$ (*N*)	*P*	2. Ambiguous cue trials				${{\rm{\chi }}}_{1}^{2}$ (*N*)	*P*
		Negative		Positive				Pessimistic		Optimistic			
		Mean	SD	Mean	SD			Mean	SD	Mean	SD		
1	Duration (s)	**0**.**43**	**0**.**15**	**0**.**35**	**0**.**15**	**9**.**86 (186)**	**0**.**0017**	0.42	0.21	0.34	0.12	3.16 (78)	0.076
2	F0 mean (Hz)	49.3	6.47	49.7	6.43	0.09 (186)	0.760	49.36	6.12	50.28	7.04	1.93 (78)	0.164
3	AM extent (dB)	4.30	3.23	3.61	2.32	1.99 (147)	0.158	3.61	2.60	4.16	3.48	0.05 (56)	0.832
4	AM rate (s-1)	3.26	1.56	3.24	1.55	0.05 (147)	0.831	3.41	1.68	2.77	1.34	2.03 (56)	0.154
5	% Time Max Intensity	46.82	16.13	50.26	15.38	1.78 (174)	0.182	44.22	13.87	51.25	14.92	3.46 (66)	0.063
6	HNR (dB)	1.79	1.78	1.79	1.57	0.37 (186)	0.545	1.82	1.47	1.77	1.66	0.41 (78)	0.520
7	Q25 (Hz)	156.34	42.05	164.44	47.05	2.75 (186)	0.097	178.76	53.42	160.49	43.46	1.06 (78)	0.303
8	Q50 (Hz)	287.82	72.49	301.4	87.57	2.17 (186)	0.140	323.11	98.60	305.84	99.08	0.01 (78)	0.927
9	Q75 (Hz)	668.79	372.9	653.38	376.8	0.41 (186)	0.524	794.21	547.94	720.97	454.32	0.03 (78)	0.858
10	F1 mean (Hz)	321.62	19.86	325.19	22.26	1.45 (185)	0.229	325.21	20.52	322.49	20.59	0.03 (78)	0.865
11	F1 range (Hz)	64.68	28.21	57.5	26.88	2.84 (185)	0.092	53.51	24.54	54.36	19.37	0.15 (78)	0.701

### Statistical analysis

Data analysis was conducted using R v. 3.0.2 (R Development Core Team 2008). The statistical analysis of the acoustic parameters was split into two analyses: (1) the trained positive/negative trials, and; (2) the ambiguous trials. This was because these two trial types, trained versus ambiguous, were fundamentally different. In the trained trials the bowls were baited with either a positive or negative stimulus and thus, were expected to elicit either a positive or negative valence state during that trial. The ambiguous trials, on the other hand, were not baited and in addition, in each of the 3 ambiguous trials, this was the first time the individual had encountred the probe in that location.

Linear mixed models were used to investigate the effect of response type (optimistic or pessimistic) on the acoustic parameters in the positive/negative trials and the ambiguous trials separately, accounting for experimental design (see electronic supplementary material for full details of models). Model residuals were visually inspected for normality and homoscedasticity. Data are presented as mean ± SD.

## Results

### Positive and negative training trials

The duration of calls produced in the negative training trials was significantly longer than the duration of calls produced during the positive training trials (raw mean ± SD: negative = 0.43 ± 0.15, positive = 0.35 ± 0.15, p = 0.0017). There was no significant difference between the positive and negative training trials in the other acoustic parameters investigated (see Table 2). To correct for multiple testing, we applied a Bonferroni correction; this led to a corrected value of p < 0.0046 (i.e. 0.05/11), which did not affect the interpretation of any of the results.

### Ambiguous trials

There were no significant effect of response type (optimistic or pessimistic) on the acoustic parameters of the grunts from the ambiguous trials (see Table 2).

## Discussion

We found that emotional valence is encoded in the duration of calls. Grunts produced during contexts of positive valence were shorter than those produced during contexts of negative valence. Call duration is affected by respiration, and changes in the action or tension of the respiratory muscles may explain the shorter duration of vocalisations in positive compared to negative contexts^10^. Longer calls produced in response to negative stimuli may function to increase the salience of these, presumably more urgent, calls to conspecifics. Previous studies in pigs have also found shorter call duration in positive contexts compared to negative contexts^28,35^, with similar findings in horses^23^, elephants^21^ and dogs^36^. Thus, call duration appears to provide a consistent indicator of emotional valence in mammalian vocalisations^10^.

Interestingly, we did not find significant differences in the other measured acoustic parameters between positive and negative contexts, which differs from recent studies^22,37,38^. There are several non-mutually exclusive explantions why the outcomes differ among studies: firstly, the emotional valence experienced by indiviudals may not have differed enough to be reflected in the measured acoustic parameters. The only difference between the two contexts was the position of the bowl and whether a reward was received or not, and the trials were carried out in quick succession. The dimensional model views valence as a continuum. Other studies may have induced emotions further apart on the continuum, whilst our experimental contexts may have induced emotions that were closer to each other on the continuum. Thus, an acoustic parameter would need to be very sensitive to subtle differences in valence to be used as an indicator of valences that are closer together on the continuum. Secondly, the experimental design of previous studies differed from ours in several other aspects, including day of recording, number of individuals present during recording and the functional relevance of the context e.g. social isolation, startling and aggression as negative contexts and food anticipation and affiliative interactions as positive contexts^22,28,37,38^. Thirdly, it may be that the calls produced here were influenced more by unmeasured factors than the valence of the contexts. Leliveld and colleagues found that the context accounted for only 1.5–11.9% of the variability in the acoustic parameters they measured^28^, suggesting that many other factors influence the acoustic characteristics of vocalisations.

Vocalisations may be more associated with the expression of arousal than valence^39,40^. Indeed, one of the main challenges in assessing emotional valence is eliciting oppositely valenced states whilst not affecting arousal, since negatively valenced states also tend to be higher arousal states^37^. For assessing the characteristics of vocalisations in such conditions, this is problematic, as high arousal is known to impact the acoustic characteristics of vocalisations^41^. Fundamental frequency parameters and energy quartiles are robust measures of emotional arousal in mammals^10,42,43^. We did not detect any statistically significant differences in these parameters between the positively and negatively valenced contexts (see Table 2). This suggests that level of arousal did not differ between the positive and negative contexts used here.

In the ambiguous trials, individuals were presented with an ambiguous cue, i.e. the bowl located in the near positive, middle and near negative locations. In contrast to the positive and negative trained trials, we did not find any significant associations between optimistic/pessimistic response type and any of the acoustic parameters measured during the ambiguous trials. However, there was a trend for calls to be shorter when produced during an optimistic response than a pessimistic response. As the training trials were rewarded/unrewarded, we predicted they would induce emotional states of positive and negative valence respectively^44^. In contrast, responses in ambiguous trials provide a measure of the animals’ underlying emotional state^14^, rather than inducing any valenced emotional state *per se*. Thus, optimistic and pessimistic responding in the ambiguous trials may not have been directly comparable to optimistic and pessimistic responding in the training trials.

Emotions are typically measured through behaviour, for example through body posture^45^, facial expression^46,47^, and vocalisations^48^. For social species, the ability to recognise the emotional state of conspecifics is likely to be adaptive as it may enable them to predict the future behaviour of the individual and adjust their behaviour accordingly^49–51^. Individuals of different species are able to perceive, and respond to, differences in emotional state based on differences in the acoustic structure of the same type of vocalisations produced in different contexts^51–54^. Changes in the characteristics of vocalisations could potentially function as a signal of emotional state to conspecifics, however further research is required to fully understand the effect of valence on acoustic parameters in animal vocalisations. Recent studies on emotional contagion suggest that cues relating to emotional state can elicit similar changes in behaviour in individuals who have no direct experience of the original cue^55–57^. Thus, changes in vocalisations corresponding with emotions could function to signal emotional state to conspecifics and to regulate social interactions.

In conclusion, duration of vocalisation appears to be a consistent indicator of valence across species^10^, and our results suggest that it may also be a sensitive indicator of small differences in valence. This is encouraging, as call duration has the advantage of being easy to measure and apply in a wide variety of contexts. To assess whether valence affects the acoustic parameters of calls, it is necessary to understand how similar or dissimilar oppositely valenced states are to each other. This could help uncover which acoustic parameters are sensitive to small changes in valence, and which others may only be affected by large differences in valence.

## Supplementary information

Supplementary methods

Related Manuscript File 1

Related Manuscript File 2

## Data Availability

The datasets generated and analysed during this study are available from the corresponding author on request.
