# Supplementary material for: Positive and negative contexts predict duration of pig vocalisations
Source: Sci Rep. 2019 Feb 14;9:2062. doi: 10.1038/s41598-019-38514-w (PMC6375976; doi:10.1038/s41598-019-38514-w)
Supplement: Supplementary file 1 — Supplementary methods [file 41598_2019_38514_MOESM1_ESM.pdf]

Positive and negative contexts predict duration of pig vocalisations

Mary Friel, Hansjoerg P Kunc, Kym Griffin, Lucy Asher, Lisa M Collins

Supplementary Methods

Selection of individuals

Overall 12 females and 24 males were selected for cognitive bias testing (12 individuals from each replicate, six per pen). On weeks 1 and 3 post-weaning, the level of injury to the body from aggression and/or injury to the tail from harmful social behaviour, was assessed and scored. To balance experimental groups, individuals were chosen based on their total injury score from weeks 1 and 3. In each pen, the two individuals with the highest injury scores, i.e. greatest number and severity of injuries, the two individuals with the lowest injury scores, i.e. lowest number and severity of injuries, and two additional individuals chosen at random from within the pen, were selected for testing.

Cognitive bias training and testing

Cognitive bias training initially involved habituating pigs to individually move from their home pens to the test pen, which was in a separate room in the same building. Once habituated to this procedure, individuals were trained to expect a positive outcome (finding three sugar coated chocolate sweets) when a bowl was placed in one corner of a test arena, and a negative outcome (finding three coffee beans) when the bowl was in the opposite corner (cf. [1] and Fig. S1). To avoid the possibility that the pigs could use olfactory cues rather than spatial cues, a false bottomed bowl was used. The false bottom contained three coffee beans and three sugar coated sweets which were inaccessible to the pigs, thus, olfactory cues were equal, regardless of location of the bowl. Coffee beans were chosen as the negative stimulus as it was found that when presented to the pigs for the first time, individuals attempted to eat the coffee beans but they were not swallowed and were expelled from the mouth. On the following presentations, individuals were found to avoid eating the coffee beans and eventually avoided the location in which they had been presented. The location of positive/negative cues was pseudo-randomly allocated and counter-balanced across the two housing environment treatments. Nine of the 36 pigs were excluded from testing due to failing to habituate to being alone in the test arena and/or eat the sugar-coated

chocolate sweets. The remaining 27 pigs that reached the testing criterion, i.e. the pig reaching the positive location within 30 seconds, *and* not approaching the negative location within 30 seconds in 8 out of 9 trials in the final training session, continued on to the test phase.

On the day of the cognitive bias test, each pig was tested individually, with the bowl in one of three intermediate probe locations (near positive, NP; middle, M; near negative, NN). These were presented once each, in a pseudo-randomized order, with the proviso that the M probe was always the first ambiguous probe presented. The ambiguous trials were administered between trials with presentations at P and N locations, resulting in nine trials per test (e.g. P, N, M, N, P, NN, P, N, NP). Ambiguous probes were unrewarded but locations P and N contained either sweets or coffee beans, as in training. In all trials, pigs were given 30 s to approach the probe after which they were returned to the start box for the next trial. Time to approach the probe was recorded from the point when all four feet were outside the start box. One experimenter operated the start box door and returned the test individual to the start box after each trial while the other experimenter recorded latency to reach the probe. Both experimenters remained out of sight during each trial to ensure that the pigs were responding only to the probe locations.

Acoustic analysis

We used PRAAT v5.3.41 DSP package for the acoustic analysis [2]. Vocalisations were visualised on spectrograms created in PRAAT (FFT method, window length = 0.025 sec, frequency steps = 250, time steps = 700, Gaussian window shape, dynamic range = 35 dB). Calls were classified as high frequency (> 500 Hz) or low frequency (< 500 Hz) [3] and only low frequency, noisy, grunt vocalisations were selected for analysis (see Table S1 for details of call sample size). All good quality vocalisations produced during the cognitive bias test, that were at least 3 calls apart if produced in a bout, were selected for analysis. All pigs were 10 weeks of age at the time of testing and vocalisation recording.

All calls were analysed using a custom-built script in PRAAT [4], which we adapted to take into account the expected vocal tract length of pigs. We calculated the expected vocal tract length of each individual by entering their weight into a regression of the nasal length on weight which was calculated from data in Garcia et al [5]. We measured source and filter related acoustic parameters, as well as duration and intensity parameters from each call. The settings to detect the F0 (pitch floor and pitch ceiling) and the formants (maximum number

of formants and maximum formant value), were set up separately for each individual based on an initial inspection of the spectrograms of their calls. More specifically, the F0 contour was extracted using a cross-correlation method (Praat settings: Time step = 0.005s, pitch floor = 30 Hz, pitch ceiling = 80 Hz). The formant analysis was conducted using linear predictive coding analysis (Praat settings: Time step = 0.0125s, maximum number of formants = 3, maximum formant = 2000 Hz, window length = 0.05s). To avoid bias between trial types, the same settings were used for all calls from an individual.

Statistical analysis

To investigate the effect of optimistic and pessimistic responses in the 'known' positive and negative contexts on acoustic parameters, we first analysed the vocalisations produced in the positive and negative training trials during the cognitive bias test. We selected vocalisations from trials in which the individual had responded correctly, i.e. in the negative training trial their response was classified as pessimistic and in the positive training trial their response was classified as optimistic. Fourteen calls were excluded from subsequent analysis due to an incorrect response and this led to a final sample of 186 calls from 23 individuals. For each acoustic parameter, we ran a separate linear mixed model (LMM: lmer function in lme4 package) including trial type (positive or negative) as the fixed effect of interest, sex and environmental treatment as fixed effects and trial number (i.e. P1, N1, N2, P2 ...) nested within pig identity as random factors to control for repeated sampling of the same individuals and trial number. To test for an effect of trial type on the acoustic parameter, for each parameter model, we compared the model with and without the fixed effect of trial type included, using likelihood-ratio tests (LRT). To do this, models were fitted with maximum likelihood estimation. The residuals were checked graphically for normal distributions and homoscedasticity. To meet the assumptions of the models. Percentage Time Max Intensity was logit transformed, AM rate, Q25, Q50 and Q75 were log transformed and AM extent was square root transformed. A GLMM with a gamma distribution and log link, with the same fixed and random factors as the LMMs, was used for duration (as the residuals from the LMM were non-normally distributed).

To test for differences in the acoustic parameters between optimistic and pessimistic responses in the ambiguous test trials we ran a separate LMM for each acoustic parameter. Each model included cognitive bias classification as the fixed effect of interest, with sex and the treatment as fixed effects, and trial (i.e. NN, NP or M) nested within individual identity

as random factors to control for repeated measures of the same individuals and trial number. To test for a significant effect of response type (optimistic or pessimistic) on the acoustic parameter, for each parameter model, we compared the model with and without cognitive bias classification included using likelihood-ratio tests. All models were fitted with maximum likelihood estimation. The residuals were checked graphically for normal distributions and homoscedasticity. To meet the assumptions of the models, Percentage Time Max Intensity was logit transformed whilst AM extent, AM rate, Q25, Q50 and Q75 were log transformed. A GLMM with a gamma distribution and log link, with the same fixed and random factors as the LMMs, was used for duration (as the residuals from the LMM were non-normally distributed).

References

1. Asher, L., Friel, M., Griffin, K. & Collins, L. M. Mood and personality interact to determine cognitive biases in pigs. *Biol. Lett.* **12,** 20160402 (2016).

2. Boersma, P. & Weenink, D. Praat: doing phonetics by computer. https://www.praat.org (2009).

3. Tallet, C., Linhart, P., Policht, R., Hammerschmidt, K., Šimeček, P., Kratinova, P., & Špinka, M.. Encoding of situations in the vocal repertoire of piglets (Sus scrofa): A comparison of discrete and graded classifications. *PLoS ONE*, **8**, e71841 (2013).

4. Reby, D. & McComb, K. Anatomical constraints generate honesty: acoustic cues to age and weight in the roars of red deer stags. *Anim. Behav.* **65,** 519–530 (2003).

5. Garcia, M., Wondrak, M., Huber, L. & Fitch, W. T. Honest signaling in domestic piglets (*Sus scrofa domesticus*): vocal allometry and the information content of grunt calls. *J. Exp. Biol.* **219,** 1913–1921 (2016).

Table S1. This table shows the number of grunts contributed by each individual to the analyses for each trial type. Only pigs that contributed at least 1 grunt to the analyses are presented ($N = 23$). The total for Positive and Negative trials combined for analysis 1 is 186 grunts. The total for the three ambiguous trials (near positive, near negative and middle) combined for analysis 2 is 78 grunts.

	Positive and Negative training trials						Ambiguous probe trials			
Pig ID	Positive 1	Positive 2	Positive 3	Negative 1	Negative 2	Negative 3	Near Negative	Near Positive	Middle	Total
**71**	1	3	2	2	3	4	3	2	1	**21**
**115**	0	2	0	0	2	3	2	0	0	**9**
**119**	2	2	1	0	0	5	1	2	2	**15**
**136**	1	2	0	0	2	3	2	1	0	**11**
**190**	3	0	2	3	3	3	2	2	2	**20**
**195**	0	0	0	0	3	0	0	0	0	**3**
**201**	2	2	1	3	3	3	0	4	1	**19**
**214**	0	0	2	0	0	3	3	2	0	**10**
**221**	0	0	1	0	0	2	0	1	1	**5**
**222**	1	2	2	0	4	3	4	2	3	**21**
**245**	0	1	0	0	1	2	0	0	1	**5**
**292**	1	1	0	2	3	3	1	0	2	**13**
**302**	0	0	0	0	3	3	2	2	1	**11**
**345**	0	0	0	0	0	2	0	0	0	**2**
**405**	1	0	1	2	2	1	1	1	0	**9**
**461**	1	0	1	0	3	0	1	0	0	**6**
**704**	1	1	3	5	3	4	1	2	1	**21**
**750**	1	1	1	2	0	3	1	2	0	**11**
**769**	0	0	1	3	2	2	2	0	1	**11**
**799**	0	0	0	0	1	1	0	1	0	**3**
**830**	0	3	2	0	4	3	3	1	1	**17**
**832**	1	0	2	0	3	4	2	1	0	**13**
**834**	0	0	0	0	2	2	2	2	0	**8**
**Total**	**16**	**20**	**22**	**22**	**47**	**59**	**33**	**28**	**17**	**264**

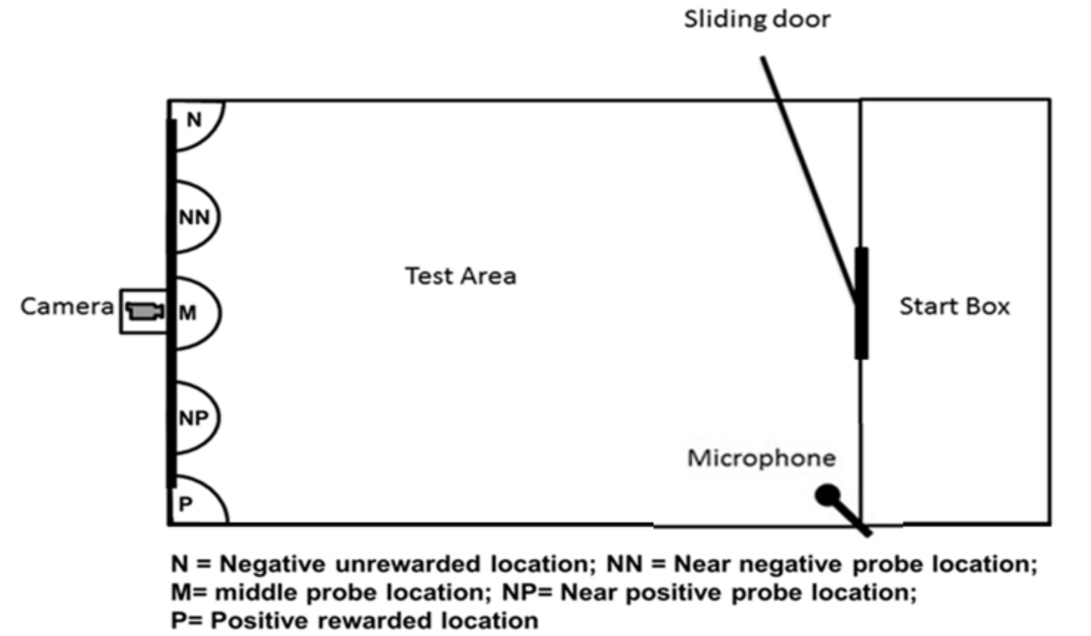

Fig. S1. Cognitive bias training and testing arena. N, negative unrewarded location; NN, near negative probe location; M, middle probe location; NP, near positive probe location; P, positive rewarded location. Note that the bowl was only present in one location per trial.
